# Genome-Wide Analyses of MADS-Box Genes in *Humulus lupulus* L. Reveal Potential Participation in Plant Development, Floral Architecture, and Lupulin Gland Metabolism

**DOI:** 10.3390/plants11091237

**Published:** 2022-05-03

**Authors:** Robert Márquez Gutiérrez, Thales Henrique Cherubino Ribeiro, Raphael Ricon de Oliveira, Vagner Augusto Benedito, Antonio Chalfun-Junior

**Affiliations:** 1 Laboratory of Plant Molecular Physiology, Plant Physiology Sector, Department of Biology, Federal University of Lavras (UFLA), Lavras 37200-900, MG, Brazil; robertmarquez993@gmail.com (R.M.G.); thalescherubino@gmail.com (T.H.C.R.); rapharicon@gmail.com (R.R.d.O.); 2 Laboratory of Plant Functional Genetics, Plant and Soil Sciences Division, 3425 Agricultural Sciences Building, West Virginia University, Morgantown, WV 26506-6108, USA

**Keywords:** ABC model, hop, transcription factors, type-II MADS box, type-I MADS-box

## Abstract

MADS-box transcription factors (TFs) are involved in multiple plant development processes and are most known during the reproductive transition and floral organ development. Very few genes have been characterized in the genome of *Humulus lupulus* L. (Cannabaceae), an important crop for the pharmaceutical and beverage industries. The MADS-box family has not been studied in this species yet. We identified 65 MADS-box genes in the hop genome, of which 29 encode type-II TFs (27 of subgroup MIKC^C^ and 2 MIKC*) and 36 type-I proteins (26 α, 9 β, and 1 γ). Type-II MADS-box genes evolved more complex architectures than type-I genes. Interestingly, we did not find FLOWERING LOCUS C (FLC) homologs, a transcription factor that acts as a floral repressor and is negatively regulated by cold. This result provides a molecular explanation for a previous work showing that vernalization is not a requirement for hop flowering, which has implications for its cultivation in the tropics. Analysis of gene ontology and expression profiling revealed genes potentially involved in the development of male and female floral structures based on the differential expression of ABC homeotic genes in each whorl of the flower. We identified a gene exclusively expressed in lupulin glands, suggesting a role in specialized metabolism in these structures. *In toto*, this work contributes to understanding the evolutionary history of MADS-box genes in hop, and provides perspectives on functional genetic studies, biotechnology, and crop breeding.

## 1. Introduction

MADS-box proteins are transcription factors (TFs) that interact with the promoters of their target genes through the binding to CArG-box *cis*-elements [1]. Phylogenetic data have classified MADS-box proteins into two groups: types I (e.g., SRF from human) and II (e.g., Mcm1 from yeast) [2]. A highly conserved sequence of about 60 amino acids called the MADS domain characterizes this family of TFs. In plants, MADS-box TFs have largely diversified and can be sub-classified into several clades. Type-I proteins are split into three groups: Mα, Mβ, and Mγ, whereas type-II proteins are classified into two groups: MIKC* and MIKC^C^ [3]. MIKC represents the protein structure of type-II MADS-box TFs, which has the conserved MADS-box for DNA-binding closed to the N-terminus followed by an intervening domain (I), a keratin-like domain (K) for protein-protein interaction, and the variable C-terminal domain. MIKC^C^ MADS-box proteins are sub-classified into 13 subfamilies, including the TM8 subfamily that is absent in *Arabidopsis* [4].

MADS-box TFs orchestrate multiple developmental programs in plants, most notably vegetative and reproductive development programs. More recently, a novel MADS-box TF in apple was implicated in regulating dormancy cycles in response to environmental cues [5]. MADS-box TFs are also involved in maintaining the spike morphology of barley under high-temperature stress [6], promoting bud break in ecodormant poplar [7], and controlling nitrogen fixation symbiosis in common beans [8]. Flowering transition is another process governed by MADS-box genes. In *Arabidopsis*, SUPPRESSOR OF OVEREXPRESSION OF CONSTANS 1 (SOC1) integrates multiple flowering signals derived from photoperiod, temperature, hormone, and age-related signals [9,10,11]. SOC1 interacts with AGAMOUS-like 24 (AGL24) and FRUITFULL (FUL) to promote flowering [12,13]. In addition, the transition from the vegetative to the reproductive phase in *Arabidopsis* is controlled by the MADS-box protein, SHORT VEGETATIVE PHASE (SVP), which is a repressor of flowering under short days [14] alike to what FLOWERING LOCUS C (FLC) does prior to vernalization [15,16]. FLC is a TF that acts as a floral repressor and is negatively regulated by cold periods or vernalization, being essential to synchronize flowering and winter [17,18]. *FLC* homologs were described in the three major eudicot lineages [19,20], including sugar-beet, apple, and coffee [19,21,22]. However, despite the role of FLC being described in the Brassicaceae [23] and more recently cereal crops and grasses [24,25], the extent to which the molecular mechanisms underlying vernalization have been conserved during the diversification of the angiosperms remains elusive.

Some of the most studied MADS-box transcription factors are involved in the development of floral organs in angiosperms [26]. Such genes are called homeotic, since their misexpression in a given whorl leads to the formation of a different floral organ [27,28,29]. This led to the formulation of the ABC model [30], which encompasses the combinatorial transcription of MADS-box TFs that elicit the developmental program of specific organs in each whorl of the flower. In this model, a class-A gene expressed alone in the first whorl leads to the formation of sepals, the co-expression of class A and B genes in the second whorl leads to the development of petals, the co-expression of class B and C genes in the third whorl elicits the formation of anthers, and finally, the expression of a C-class gene alone in the fourth whorl leads to the formation of carpels.

Further research extended the ABC model to an ABCDE model, where D-class genes expressed in the carpel lead to the formation of ovules, and E-class genes expressed in all whorls form tetramers with ABC TFs that coordinate the development of each whorl [1,31]. In *Arabidopsis*, the ABCDE model encompasses the A-class APETALA1 (AP1) [32]; the B-class APETALA3 (AP3) and PISTILLATA (PI) [33,34]; the C-class AGAMOUS (AG) [35]; the D-class AGAMOUS-like 1, 5, and 11 (AGL1, AGL5, and AGL11) [36]; and the E-class SEPALLATA 1,2,3,4 (SEP1, SEP2, SEP3, and SEP4) [37]. The ABCDE model has been conserved throughout angiosperm evolution. The genes encompassing each homeotic class have been determined in different species, from monocots, such as rice [38,39], wheat [40], and Easter lily [28]; to dicots, such as soybean [41,42], coffee [22], and the New Zealand endemic species, *Clianthus maximus* [43], among many others. Moreover, studies have also revealed the significance of type-I MADS-box transcription factors in plant reproduction [44].

Hop (*Humulus lupulus* L.) is a perennial crop that belongs to the Cannabaceae family. It blooms in short days once it develops a particular number of nodes. It has been phenotypically demonstrated that vernalization and dormancy do not influence flower yield and quality [45,46]. Moreover, hop is an economically important species because its cones (female inflorescences) are widely utilized in the pharmaceutical and beer industries [47,48,49]. However, the mechanisms involved in the reproductive phase transition and flower development remain poorly explored at the molecular genetics level. We carried out a genome-wide approach, identified 65 MADS-box genes in the hop genome, and further studied their phylogenetic relationships, genetic structure, and gene expression profiles using publicly available RNA-Seq data. This study revealed TFs that potentially coordinate critical aspects of plant development, phase transition, and glandular metabolism. Therefore, this work advances our understanding of the evolutionary history of the MADS-box TFs in hop and opens new avenues for functional genetic research and crop breeding toward expanding its production zones in the world, especially in the tropics.

## 2. Results

### 2.1. MADS-Box Genes Encoded in the Hop Genome and Gene Ontology Annotation

Using AUGUSTUS on RNA-Seq libraries, our bioinformatics pipeline identified 47 genes coding for proteins with canonical MADS-box domains. Moreover, the hop genome sequence has 69 genes annotated as coding for MADS-box proteins. The overlap of our results with the official hop genome annotation shows a set of 65 non-redundant genes (unigenes: HlMADS01 to HlMADS65), seven of which exclusively from our prediction, 23 exclusively in the hop genome annotation, and 35 represented in both sets. All seven novel genes identified in our *de novo* prediction pipeline were MIKC^C^-type proteins (HlMADS28-33 and HlMADS65). The encoded protein length ranged from 135 to 547 amino acid residues (aa), with an average of 249 aa; the molecular mass varied from 16 to 60 kDa, and the isoelectric point was between 4.55 and 10.25 (Table S1).

Gene Ontology (GO) analysis was performed on the 65 hop MADS-box proteins with the Blast2GO software (Table S3). All hop MADS-box proteins were classified into the three main categories (cellular component, molecular function, and biological process) and their subcategories (Figure S6). In this analysis, 48 proteins returned for cellular component and further split into six subgroups, being the ‘cell part, cell, and organelle’ subcategory the most over-represented subgroup (74%), which is a share even greater than that of *Arabidopsis* (56%), according to our analyses. In addition, 1.5% of the hop MADS-box proteins were annotated into the membrane subcategory, in contrast with 2.8% in *Arabidopsis.* On the other hand, all 65 hop MADS-box proteins were annotated in the subcategory binding of the category molecular function, a result identical to *Arabidopsis*. The second most represented molecular function subcategory in the hop MADS-box protein set was transcription regulator activity, with 75% of the proteins, in contrast with 87% for *Arabidopsis*. The subcategory catalytic activity did not contain any hop MADS-box proteins. Finally, the category biological process contained 51 hop MADS-box proteins (79%). The subcategories biological regulation, cellular processes, regulation of biological processes, and metabolic process were the most overrepresented (97.5%) in hop, followed by positive regulation of biological process subcategory (92.5%). All other subcategories were represented by less than 20% of the hop MADS-box protein set.

### 2.2. Phylogenetic Analyses Revealed Clades and Potential Functions of Hop MADS-Box Genes

**Figure 1:**
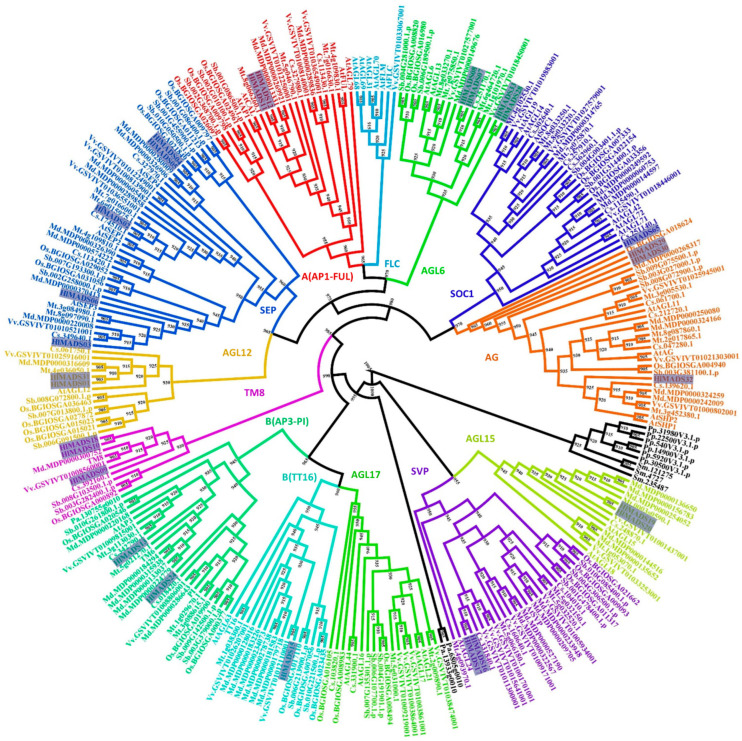
Phylogenetic tree of MIKC^c^-type MADS-box proteins of *Humulus lupulus* ((Hl) (27 sequences, highlighted)), *Arabidopsis thaliana* ((At)(39)), *Solanum lycopersicum* (TM6 and TM8), *Physcomitrella patens* ((Pp) (6)), *Selaginella moellendorffii* ((Sm) (3)), *Piceaabies* ((Pa) (3)), *Sorghum bicolor* ((Sb) (32)), *Oryza sativa* subsp. *indica* ((Os) (32)), *Cucumis sativus* ((Cs) (26)), *Malus domestica* ((Md) (42)), *Medicago truncatula* ((Mt) (35)), and *Vitis vinifera* ((Vv) (33)). Sequences from *Selaginella moellendorffii, Physcomitrella patens*, and *Piceaabies* were used as outgroups (in black font).

**Figure 2:**
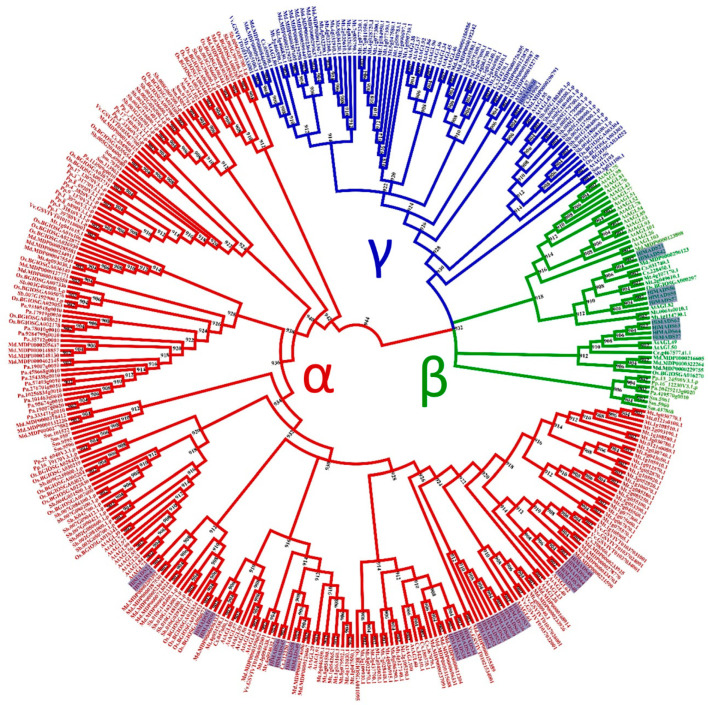
Phylogenetic tree of I-type MADS-box proteins of *Humulus lupulus* ((Hl) (36 sequences, highlighted)), *Arabidopsis thaliana* ((At) (58)), *Chlamydomonas reinhardtii* ((Cr) (1)), *Physcomitrella patens* ((Pp) (16)), *Selaginella moellendorffii* ((Sm)(14)), *Piceaabies* ((Pa) (18)), *Sorghum bicolor* ((Sb) (38)), *Oryza sativa* subsp. *indica* ((Os) (36)), *Cucumis sativus* ((Cs) (12)), *Malus domestica* ((Md) (56)), *Medicago truncatula* ((Mt) (79)), and *Vitis vinifera* ((Vv) (10)).

Protein domain analyses identified 29 type-II and 36 type-I MADS-box proteins encoded in the hop genome. Each set was submitted to separate phylogenetic analyses to further classify them into subfamilies (Figure 1, Figure 2 and Figure S1). Our results revealed five members in the SEP clade, two in the A clade (AP1-FUL), two in the B clade (AP3-PI), three in the C/D clade (AG), four in the AGL6, two in the AGL12, two in the AGL15, three in the TM8, one in the BS (TT16), two in the SVP, and one in the SOC1 clade (Figure 1, Table S1). Two type-II proteins were classified as MIKC* (Figure S1). Remarkably, the FLC and AGL17 subfamilies are not represented in the hop genome. The only member of the SOC1 subfamily (*HlMADS65*) was found when a tBLASTn was performed using the *Arabidopsis* SOC1 protein sequence as a query, and AUGUSTUS was run on the genome region identified in the output. *HlMADS65* lies within the region annotated as an intron of *000453F.g47* (Figure S2). The same approach was used for other genes, resulting in no new sequences.

For the type-I MADS-box proteins, the α-subfamily encompasses the most represented group, with 26 members, followed by the β-subfamily (nine members). In contrast, the γ-subfamily is represented by a single member (Figure 2).

### 2.3. Structural and Motif Analyses of Hop MADS-Box Genes

**Figure 3:**
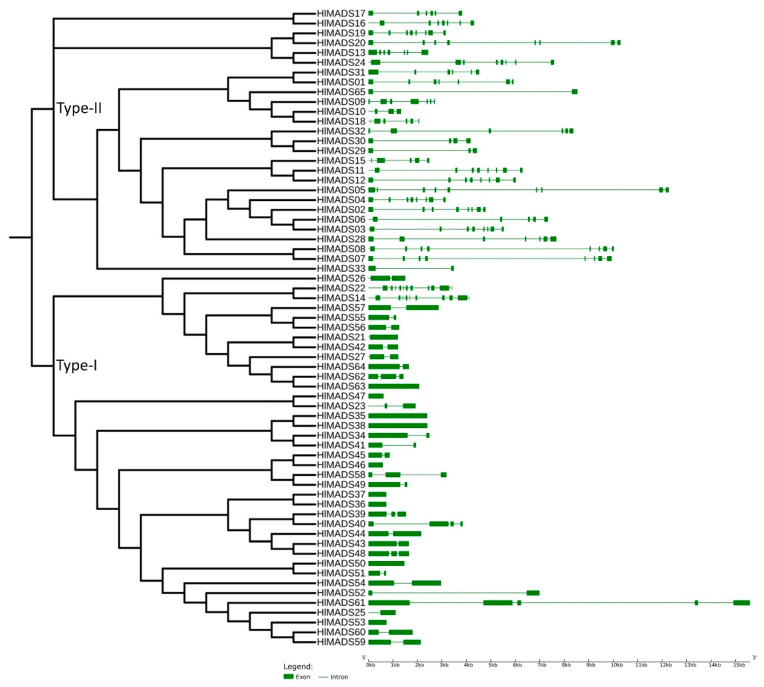
Phylogenetic tree and structure of 65 hop MADS-box genes. Exons are represented by green solid boxes, and introns by green lines.

To confirm our phylogenetic relationships and gain further insights into gene functions, we explored the exon-intron architecture of MADS-box genes. The number of exons among the 29 type-II MADS-box genes varied from 2 (*HlMADS33* and *HLMADS65*) to 13 (*HlMADS22*, the sole member of the MIKC* group). In hop, *HlMADS05* is the longest type-II MADS-box gene (12.5 kb), with nine exons and eight introns. In the type-I group, the exon number varied from one to five. Overall, *HlMADS61* was the longest gene (16 kb), whereas the shortest ones were *HlMADS46* (584 bp) and *HlMADS47* (613 bp), both with only one exon each (Figure 3), all falling in the type-I clade.

**Figure 4:**
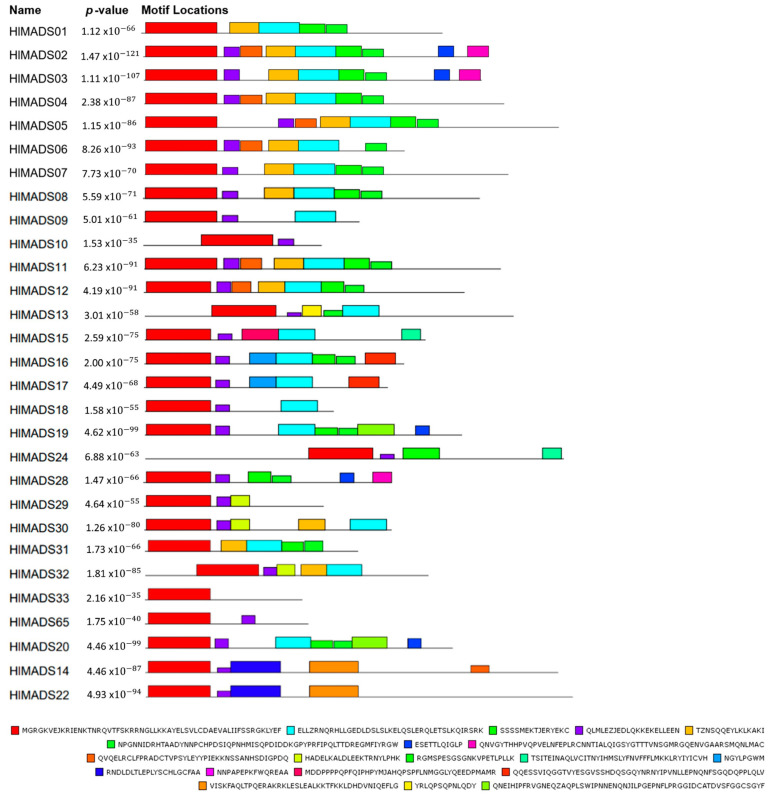
Motif distribution of hop type-I MADS-box proteins. Protein sequences are represented by black lines, and the conserved motifs are represented by colored boxes with the MADS-box domain in red.

The MEME tool was used to visualize conserved protein domains. While all type-II proteins displayed the MADS-box domain in the tool output, only 21 proteins had the K-box domain, even though this motif is a characteristic feature of this group (Figure 4). However, the NCBI’s Conserved Domain Search tool resulted in 23 proteins with the K-box domain, consequently adding HlMADS28 and HlMADS29 to the list. Even though the MADS domain did not appear for HlMADS55, HlMADS56, and HlMADS57, in the MEME analyses, the domain presence was confirmed by NCBI and PFAM conserved domain inference tools. All I-type proteins displayed the MADS domain in MEME but at diverse locations of the peptide sequence (Figures S3 and S4).

### 2.4. Transcriptional Profile of Hop MADS-Box Genes in Different Tissues

**Figure 5:**
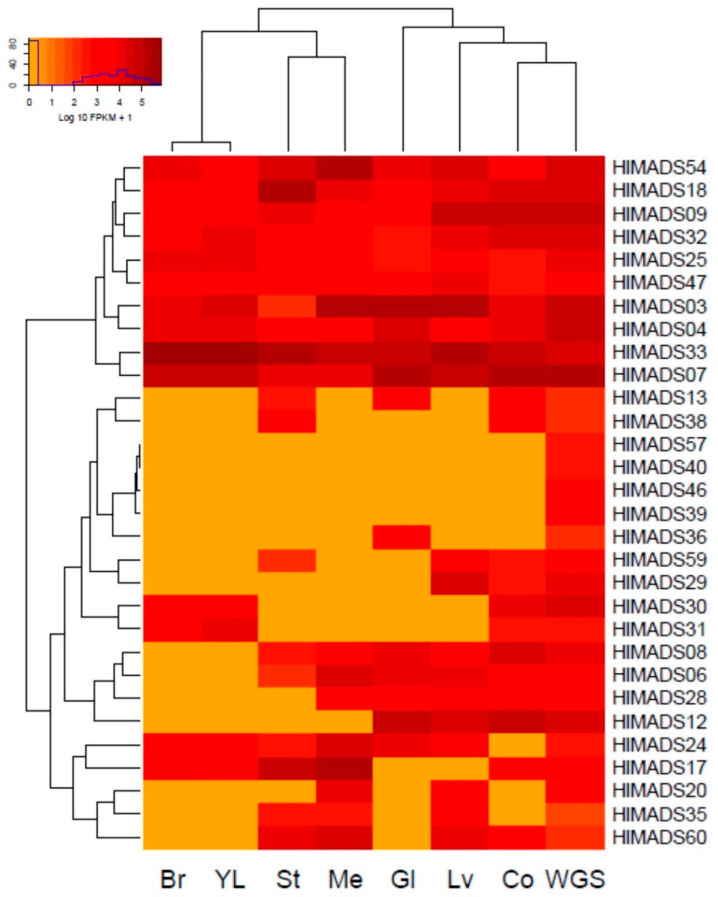
Expression profiling of hop MADS-box genes in RNA-Seq libraries: bracts (Br), young leaves (YL), stem (St), vegetative meristem (Me), glands (Gl), mature leaves (Lv), cones without glands (Co), and a mélange of plant tissues collected throughout the whole growing season (WGS).

The expression profiling of MADS-box genes in different hop tissues was determined through the analysis of publicly available RNA-Seq data deposited in the NCBI-SRA database. Thirty genes were expressed in different samples (Figure 5), whereas 35 were not expressed. Of the expressed genes, 18 are of the MIKC^C^ type, and 12 are type I. Some genes belonging to the same subfamily showed distinct expression patterns. For example, in the SEP subfamily, *HlMADS03* and *HlMADS04* were expressed in all samples analyzed, whereas *HlMADS06* was expressed only in the stem, meristem, glands, mature leaves, and cones without glands. Interestingly, young leaves and bracts showed an identical expression pattern for the MADS-box genes. Moreover, *HlMADS17*, *HlMADS30*, and *HlMADS31* (respective members of the SVP, C/D(AG), and AGL12 clades) were expressed in young leaves compared with the mature organ, indicating a possible function during leaf development and expansion.

We identified two gene groups regarding the expression profile (Figure 5). The first encompassed constitutive genes: two SEP (*HlMADS03* and *HlMADS04*), two TM8 (*HlMADS09* and *HlMADS18*), two AGL6-like (*HlMADS07* and *HlMADS33*), one of the C/D (AG) subfamily (*HlMADS32*), and three type-I α (*HlMADS25*, *HlMADS47*, and *HlMADS54*). The second group included 20 genes (9 type-I and 11 MIKC^c^) that showed variable expression among the samples. The B (AP3-PI) subfamily member, *HlMADS13*, was expressed in the stem, glands, and cones, whereas four type-I genes (*HlMADS57*, *HlMADS40*, *HlMADS46*, and *HlMADS39*) were expressed only in the whole plant sample. Interestingly, a type-I α gene, *HlMADS36*, was expressed only in the glands. On the other hand, two C/D(AG) subfamily genes (*HlMADS29* and *HlMADS30*) are in this group, with *HlMADS29* being expressed in mature leaves and cones, while *HlMADS30* expressed in the bracts and young leaves, similar to *HlMADS31*. Meanwhile, *HlMADS06* (SEP) and *HlMADS08* (AGL6) did not express in bracts or young leaves; *HlMADS28* (AGL6) expressed in the meristem, glands, mature leaf, and cones; *HlMADS12* [A(AP1-FUL)] expressed in glands, leaf, and cones; *HlMADS24* [B(AP3-PI)] expressed in all samples but cones without glands. In turn, *HlMADS17* (SVP) was expressed in all samples but glands and mature leaves, whereas *HlMADS20* was expressed only in the meristem and mature leaves. Lastly, *HlMADS35* and *HlMADS60* (type-I α-subfamily) expressed in the stem, meristem, and mature leaves, while *HlMADS60* expressed in cones, and *HlMADS35* did not.

## 3. Discussion

Many MADS-box proteins function as master regulatory transcription factors controlling critical aspects of plant development and agricultural traits. The genomic characterization of this gene family has been carried out for several plant species, such as *Arabidopsis* (107 genes: [50]), rice (75 genes: [51]), grapevine (74 genes: [52]), *Pyrus* (75 genes: [53]), to name a few. This work identified and provided the transcriptional profiling of 65 MADS-box genes in the hop (*H. lupulus* L.) genome. First, we carried out a *de novo* gene annotation of the hop genome and combined it with the official one available on the HopBase platform [54]. This pipeline identified seven novel MADS-box genes (*HlMADS28-33* and *HlMADS65*) in the hop genome with reads from RNAseq libraries aligned on these genes (Figures S2 and S7). Additionally, it was possible to find some genes (i.e., *HlMADS01* and *HLMADS02*) which were only identified when AUGUSTUS was run with the UTR parameters turned off. This finding shows that *de novo* gene prediction outputs, as well as the official functional genome annotation, must be interpreted with caution.

In our phylogenetic analyses, 27 MADS-box proteins were classified into 11 clades based on their relationships with the *Arabidopsis* MIKC^C^-type and TM8 subfamilies. We discovered that the AGL17 subfamily is not represented in the hop genome, similarly to pears [53]. Neither the FLC subfamily is represented in the hop genome, suggesting that the species lacks the vernalization route completely. Accordingly, it has been demonstrated that hop does not require vernalization to trigger flowering [45], but instead, the process must involve other induction routes, such as photoperiod and age [46].

According to the photoperiod route in *Arabidopsis*, SOC1 integrates photoperiod signals to promote flowering under long-day conditions [55]. It was possible that in hop, a functional homolog was similarly involved in flowering induction. However, no SOC1 ortholog was identified in our gene prediction pipelines. Notwithstanding, the HopBase genome annotation includes a gene (*000453F.g47*) that contains only the K-box domain characteristic of the SOC subfamily. That may be the reason our BlastP analysis using the MADS-box consensus sequence as the query did not identify this gene in the hop proteome. However, the MADS domain for this gene was recognized by AUGUSTUS in the first annotated intron of *000453F.g47*, where RNA-Seq reads aligned (Figure S2). This gene displays two exons and encodes a protein with the MADS domain but without the K-box, suggesting that it was incorrectly annotated or may undergo intron retention, which is the most predominant mechanism of alternative splicing in plants [56]. Moreover, two SVP subfamily genes were identified in the hop genome, with *HlMADS17* expressed in young leaves only, indicating potential participation in leaf development and expansion. Finally, two AGL15 subfamily genes (*HlMADS19* and *HlMADS20*) were identified, with the latter expressed in mature leaves and the meristem. In hop, these genes could act as repressors of flowering transition since members of this subfamily act as flowering repressors in *Arabidopsis* [57] and promote the expression of miR156, a *bona fide* marker of plant juvenility [58].

Our phylogenetic study also identified two AP1-FUL subfamily genes (*HlMADS11* and *HlMADS12*) in the hop genome. In *Arabidopsis*, AP1 determines floral meristem identity and, later, also in petal and sepal development [32,59]. The expression of *HlMADS12* in hop cones suggests a possible participation in determining floral organ identity (Figure 5). Even though female flowers of hop (cones or strobiles) entirely lack the perianth (sepals and petals), male flowers have sepals [60], thus showing that the A function of the ABC model of flower development is present in hop. Another hypothesis for the lack of perianth structures in female hop flowers could be a lack of the E-class (SEP subfamily) function. However, this subfamily is the most represented within the MIKC^C^ clade in this species, with five genes expressed in different tissues (Figure 5). The repression mechanism of the A- and B-functions in the first two whorls during cone development remain to be ascertained. Figure 1 shows the B-class *HlMADS13* was closest to AP3 while *HlMADS24* was closest to PI. These genes showed distinct gene structures, with *HlMADS24* being longer than *HlMADS13*, containing an extra exon (Figure 3), and lacking the K-box (Figure 4). When comparing GO terms between *HlMADS13* and *HlMADS24* (Table S3), both displayed the same terms, results which are comparable for the *Arabidopsis* AP3 and PI. Therefore, it is plausible that the B-function in hop is conserved during flower development.

Dioecious reproduction is a common feature found in the Cannabaceae, including *Cannabis* and *Humulus*. The organ positions in male (staminate) and female (pistillate) hop flowers deviate from the prevalent four-whorl scheme (sepals-petals-stamens-carpels) observed in many angiosperm groups [60]. After the shoot meristem undergoes flowering transition, the formation of floral organs is initiated. Male and female inflorescence meristems are distinguishable at the anatomical level very early on [60], when the program of floral organ developmental fate has already been decided. According to the classic ABC model, also known as the ABCDE model, the expression of class A and C genes are mutually exclusive in the floral meristem [30]. Three C/D-class genes were identified in the hop genome (Figure 1). The presence of sepals in the first of the two whorls of the male flower suggests a partial presence of the A-class function. In contrast, the development of stamens in the second whorl reveals the expression of B and C class genes and repression of the class A gene function. On the other hand, in female flowers (cones), the formation of a rudimentary perianth (without the development of sepals or petals) indicates an absence of ABC gene expression. In contrast, the development of two carpels in its second whorl indicates the exclusive expression of the C function. Finally, *HlMADS29* probably carries out the D-class function because it is expressed in the cones (Figure 5) and its gene annotation is associated with ovule development (GO:0048481). A more refined definition of gene expression within the developing flower and functional analyses to define the role of ABCDE genes are warranted to better understand the genetics of floral organ development in this species.

The 36 type-I MADS-box genes identified in the hop genome were classified into three subfamilies, α (26 genes), β (9), and γ (1). These genes were more structurally diverse but contained fewer exons than the type-II genes (Figure 3). Previous research reported similar results in other species [50,53,61]. The amino acid sequence in this group was also more diverse than in the type-II group, and the MADS-box was not detected in some analyses for three members of the β-subfamily (*HlMADS55*, *HlMADS56*, and *HlMADS57*), although they were present when manually inspected (Figure S4). The MADS motif is somewhat divergent in these three proteins and required a higher sensitivity from the sequence analysis tool. According to the gene expression profile, 12 type-I *MADS-box* were expressed in the RNA-Seq libraries analyzed, with *HlMADS57* the only member of the β-subfamily to be expressed. Interestingly, *HlMADS36* was exclusively expressed in all three gland samples analyzed (Figure S5), which allowed us to hypothesize that it may coordinate the biosynthesis of resin and specialized metabolites [62] or participate in gland development. Therefore, it is important to further verify the exact timing expression domain and function of *HlMADS36*. Moreover, *HlMADS36* is associated with a GO term (0045944) involved in multiple processes related to transcriptional induction of genes related to the metabolism of organic compounds. Overall, our findings provide perspectives on functional analyses and breeding of hop.

## 4. Materials and Methods

### 4.1. Gene Prediction

*H. lupulus* L. gene prediction was performed using AUGUSTUS version 3.3.3 [63]. RNA-seq libraries retrieved from the NCBI’s SRA database guided the proper identification of exon-intron gene boundaries (Table S2; accessed on 7 January 2020). After quality evaluation with FastQC, the libraries were processed with Trimmomatic v.0.39 [64] to remove adapter sequences and fragments with poor overall Phred quality. High-quality libraries were then aligned to the *H. lupulus* L. masked genome sequence from the HopBase platform [54] (accessed on 7 January 2020) using HISAT2 v.2.1 [65]. During the training phase to establish AUGUSTUS metaparameters for the species, RNA-seq libraries from glands, leaf, cones without glands, and meristem (SRR575195, SRR10589377, SRR575201, SRR10320794, respectively) were assembled using Trinity v.2.11.0 [66]. Candidate coding regions were identified with TransDecoder v.5.5.0 [47]. Subsequently, protein sequences were generated and utilized to train AUGUSTUS according to Alternate protocol 1 [67]. The training was also enriched with EST and UTR coordinates utilizing coding sequences from Trandecoder and the PASA pipeline [68]. Sorted BAM files were used to generate exon (with Bam2wig) and intron hints. Finally, the trained metaparameters were fed into AUGUSTUS for gene prediction.

### 4.2. Identification of MADS-Box Genes in the Hop Genome

We used the Basic Local Alignment Search Tool BLAST v.2.11.0 [69] to scan the hop proteome searching for MADS-box proteins. A conserved domain sequence from Serum Response Factor (SRF) retrieved from the Pfam database (http://pfam.xfam.org/, accessed on 14 May 2021) was used as a query in BlastP against proteins obtained previously in the gene prediction step. In parallel, BlastP was carried out against the hop proteome retrieved from the HopBase platform. We only considered MADS-box proteins with sequences presenting a conserved domain with all three Pfam, SMART, and NCBI-BlastP analyses. Redundant proteins reported on the same locus were combined after manually curating genomic loci with IGV. Putative MADS-box protein sequences with less than 100 amino acid residues were re-submitted to AUGUSTUS, with UTR parameters turned off, until new sequences were no longer retrieved. Each non-redundant, putative MADS-box protein sequence identified in these analyses was named HlMADS01 to 65. Their physicochemical properties (length of amino acid sequence, molecular weight, and isoelectric point) were determined with the ExPASy Proteomics tool (https://web.expasy.org/protparam/, accessed on 14 May 2021).

### 4.3. Phylogenetic Analysis

MADS-box protein sequences retrieved from species spanning the plant kingdom *(Chlamydomonas reinhardtii, Physcomitrella patens, Selaginella moellendorffii, Piceaabies, Sorghum bicolor, Oryza sativa, Cucumis sativus, Malus domestica, Medicago truncatula,* and *Vitis vinifera*) were retrieved from the PlantTFDB database v.5.0 [70] along with those from *Arabidopsis thaliana* and *Solanum lycopersicum* retrieved from the NCBI database, along with those from H. lupulus identified above, were used in our phylogenetic analysis. For sequence types I, MIKC^c^, and MIKC*, multiple sequence alignment jobs were performed separately with MAFFT v.7.475 [71]. The alignment quality was evaluated with GUIDANCE 2 v.2.02 [72]. Both steps used default parameters. Phylogenetic trees were inferred with PHYLIP v.3.696 [73] with 1000 bootstrap replicates, using the Jones-Taylor-Thornton matrix and neighbor-joining method [74]. Finally, the tree was visualized with FigTree, and the hop MADS-box proteins were classified into subgroups according to the *Arabidopsis* MADS-box subfamilies [50] plus the subfamily TM8 first reported in tomato [4]. When a subfamily was absent in a first search, tBLASTn was performed using the protein sequence from *Arabidopsis* of that subfamily as the query.

### 4.4. MADS-Box Gene Structure and Conserved Protein Motif Analyses in Hop

The exon-intron structures of MADS-box genes were identified with the Gene Structure Display Server GSDS2.0 [75] using the GFF files generated from our gene prediction as well as the annotation available at the HopBase. The MEME suite online analysis tool [76] was used to identify putative motifs of hop MADS-box proteins with the following parameters: maximum of 20 motifs to be identified and motif width between 6 and 60. In this case, we used sequences from *Chlamydomonas reinhardtii*, *Physcomitrella patens*, *Selaginella moellendorffii*, *Picea abies*, *Sorghum bicolor*, *Oryza sativa*, *Cucumis sativus*, *Malus domestica*, *Medicago truncatula*, and *Vitis vinifera*, as background normalization. Finally, the conserved motifs obtained were verified with PFAM, SMART, and NCBI conserved domain inference tools.

### 4.5. Gene Ontology (GO) Annotation of the Hop MADS-Box Genes

The MADS-box genes of hop were annotated into each of the three categories of Gene Ontology (GO: biological process, molecular function, and cellular component) using the Blast2GO software [77], and the results were visualized with WEGO [78].

### 4.6. Expression Analysis of MADS-Box Genes in Hop Tissues

Gene transcription profiling of hop MADS-box genes was generated with NCBI-SRA RNA-seq libraries of meristems (SRR10320793), stems (SRR10320795), leaves (SRR575205), young leaves (ERR2040411), cones without glands (SRR575201), bracts (SRR10541757), glands (SRR575193), and a sample of the whole plant during the growing season (SRR4242068). The data were aligned to the hop masked genome sequence with STAR v.2.7.7 [79] using default parameters. The number of aligned reads was quantified with the htseq-count function in HTseq v.0.11.5 [80] assuming no strand specificity. The quantified reads were normalized as FPKM (Fragments Per Kilobase of transcript per Million mapped reads) with the edgeR package. Finally, a heatmap of MADS-box gene expression was generated in R v.3.6.3 using the gplots package. To corroborate these results, we used Samtoolsto filter all reads uniquely mapped to the genome. Subsequently, each locus was visualized with IGV, and the alignments for selected genes are reported in Figure S7.

## 5. Conclusions

In this work, we identified 65 MADS-box genes in the hop genome, with 36 being of type I and 29 genes of type II. Phylogenetic analyses showed that 27 type-II MADS-box genes belonged to 12 subfamilies, while two genes were of type MIKC*. Meanwhile, type-I MADS-box genes were classified in α-subfamily (26 members), β-subfamily (nine members), and γ-subfamily (a single member). The gene structure of type-I genes was less complex than that of type II genes, with fewer exons, even though the longest MADS-box gene was of type I. Some MIKC^C^-type MADS-box proteins did not display the K-box domain. Members of the FLC subfamily were not found in the hop genome. The only SOC1 subfamily member in the hop genome may undergo alternative splicing with intron retention. Genes of the ABCDE model of flower development were expressed in cones. One gene, a member of the α-subfamily, was found exclusively expressed in lupulin glands, with potential implications for specialized metabolism. Thus, this work contributes to understanding the evolutionary history of MADS-box in hop and provides perspectives on functional analysis and crop breeding.

## Data Availability

Main data supporting the findings of this study are available in the manuscript and online supplementary materials. The raw data used for the analyses and figures are available upon request to A.C.-J.

## Supplementary Materials

The following supporting information can be downloaded at: https://www.mdpi.com/article/10.3390/plants11091237/s1, Table S1: MADS-box genes identified in the hop genome. Table S2: RNA-seq libraries used to guide AUGUSTUS gene prediction and transcriptional profiling. Table S3: Gene Ontology terms of the hop MADS-box gene family. Figure S1: Phylogenetic tree of MIKC*-type MADS-box proteins of *H. lupulus* (2, in red font)*, Arabidopsis* (6, in black font). Figure S2: RNA-Seq reads aligned on the gene *000453F.g47*. Figure S3: Motif distribution of type-I MADS-box proteins in hop. Protein sequences are represented by black lines, and the conserved motifs are represented by colored boxes. Figure S4: Motif distribution of hop β-subfamily MADS-box proteins. Protein sequences are represented by black lines, and the conserved motifs are represented by colored boxes. (**A**) Logo of MADS-box domain. (**B**): Motif distribution of the hop β-subfamily MADS-box proteins. (**C**) Amino acid residues within MADS-box domains. Figure S5: Expression profile of the hop MADS-box genes in three RNA-Seq libraries from cones, leaves, and glands. Figure S6: Gene Ontology classification of MADS-box genes in hop and *Arabidopsis*. Figure S7: RNAseq reads that are uniquely mapped on six novel MADS-box genes (HlMADS28-33).
