# Supplementary material for: Genome-Wide Analyses of MADS-Box Genes in Humulus lupulus L. Reveal Potential Participation in Plant Development, Floral Architecture, and Lupulin Gland Metabolism
Source: Plants (Basel). 2022 May 3;11(9):1237. doi: 10.3390/plants11091237 (PMC9100628; doi:10.3390/plants11091237)
Supplement: Supplementary file 1 [file plants-11-01237-s001.zip › plants-1670456-supplementary.pdf]

Table S1. *MADS-box* gene identified in the hop genome.

Gene ID	Gene name	Contig Location	CDS (bp)	Exon No.	Strand	Protein size (residues)	MW (Da)	pI	Subfamily
**g126639.t1**	HlMADS01	005050F:261365:267689	648	7	-	215	24191.15	8.66	AGL12
**g4287.t1**	HlMADS02	000025F:2663950:2669377	747	9	-	248	28673.47	8.96	E(SEP)
**000727F.g16.t1**	HlMADS03	000727F:300503:306308	732	8	+	243	28010.83	8.78	E(SEP)
**000079F.g81.t1**	HlMADS04	000079F:2009261:2019723	780	8	+	259	29495.61	7.57	E(SEP)
**001022F.g25.t2**	HlMADS05	001022F:835353:848110	897	9	-	298	34337.21	7.57	E(SEP)
**006765F.g2.t1**	HlMADS06	006765F:49016:57362	567	7	+	188	22130.51	9.68	E(SEP)
**000139F.g16.t1**	HlMADS07	000139F:691180:701232	843	8	-	262	30480.39	8.73	AGL6
**000261F.g24.t1**	HlMADS08	000261F:1206883:1217085	729	8	+	242	28455.11	8.87	AGL6
**000155F.g57.t2**	HlMADS09	000155F:1174916:1179017	828	9	-	156	17770.36	9.45	TM8
**004947F.g24.t1**	HlMADS10	004947F:296392:297739	477	4	+	129	14846.84	9.37	TM8
**006412F.g5.t1**	HlMADS11	006412F:101506:107824	774	8	+	257	29984.02	8.2	A(AP1-FUL)
**005516F.g3.t1**	HlMADS12	005516F:100575:106871	771	8	-	256	29778.06	8.97	A(AP1-FUL)
**000562F.g29.t1**	HlMADS13	000562F:405065:407961	930	7	-	295	33152.62	6.55	B(AP3-PI)
**000267F.g44.t1**	HlMADS14	000267F:1322662:1327001	1026	11	+	341	38315.62	6.26	MIKC*
**000006F.g39.t2**	HlMADS15	000006F:1630741:1633244	654	5	+	225	25689.19	7.07	BS(TT16)
**008535F.g2.t2**	HlMADS16	008535F:35736:40789	628	9	+	208	23976.43	6.55	SVP
**003895F.g10.t1**	HlMADS17	003895F:119273:124319	588	7	-	195	22415.69	6.86	SVP
**004012F.g22.t2**	HlMADS18	004012F:362046:365098	519	8	+	152	17407.84	9.3	TM8
**002163F.g38.t1**	HlMADS19	002163F:484371:488562	765	9	-	254	28867.72	8.23	AGL15
**g128133.t1**	HlMADS20	005238F:252962:256110	597	8	-	254	28872.72	8.24	AGL15
**004180F.g12.t1**	HlMADS21	004180F:187393:188704	1140	1	+	379	42912.16	6.83	Beta
**000351F.g15.t1**	HlMADS22	000351F:597580:601145	1062	13	+	353	40110.17	6.61	MIKC*
**000962F.g16.t1**	HlMADS23	000962F:609956:612029	603	3	+	200	22140.06	5.75	Alpha
**000586F.g19.t1**	HlMADS24	000586F:511047:518658	1008	8	+	335	38534.19	8.84	B(AP3-PI)
**000238F.g55.t1**	HlMADS25	000238F:849739:851157	621	2	+	206	23333.47	6.08	Alpha
**001957F.g8.t1**	HlMADS26	001957F:323596:325350	1338	2	+	445	48673.45	9.02	Gamma
**000887F.g29.t1**	HlMADS27	000887F:890609:891924	915	2	+	304	35110.43	5.25	Beta
**g39417.t1**	HlMADS28	000628F:1010162:1017835	597	7	-	198	22484.65	8.94	AGL6
**g90065.t1**	HlMADS29	002424F:490038:494465	435	3	-	144	17011.7	9.6	C/D(AG)
**g21585.t1**	HlMADS30	000253F:1451643:1455810	597	4	-	198	23006.82	9.7	C/D(AG)
**g98509.t1**	HlMADS31	002879F:359907:364430	531	6	-	176	20176.59	8.77	AGL12
**g57516.t1**	HlMADS32	001131F:491536:499904	705	6	-	234	26967.13	9.91	C/D(AG)
**g120931.t1**	HlMADS33	004517F:301077:304561	393	2	+	130	15020.97	10.25	AGL6
**g105746.t1**	HlMADS34	003321F:169727:172210	630	2	-	209	22689.95	8.91	Alpha
**g126627.t1**	HlMADS35	005050F:116796:119190	594	1	+	197	21541.38	7.71	Alpha
**g145841.t1**	HlMADS36	008392F:89967:90690	585	1	-	194	22721.07	8.63	Alpha
**g115481.t1**	HlMADS37	004059F:231977:232700	585	1	-	194	22663.03	8.84	Alpha
**g98489.t1**	HlMADS38	002879F:155406:157810	627	1	+	208	22639.82	9.18	Alpha
**g70848.t1**	HlMADS39	001574F:102007:103540	990	3	-	329	36408.27	9.79	Alpha
**g148256.t1**	HlMADS40	009280F:58747:62590	867	4	-	288	31504.4	9.49	Alpha
**g63684.t1**	HlMADS41	001329F:293116:295050	573	2	+	190	20693.75	9.61	Alpha
**g116966.t1**	HlMADS42	004180F:181006:182210	960	2	+	319	35943.8	8.58	Beta
**g115485.t1**	HlMADS43	004059F:341647:343290	1297	2	-	431	46701.58	9.36	Alpha
**g115484.t1**	HlMADS44	004059F::322287:324440	1644	2	-	547	60026.5	9.46	Alpha

**g143617.t1**	HlMADS45	007779F:47307:48170	459	2	-	152	17620.07	9.69	Alpha
**g93707.t1**	HlMADS46	002616F:159796:160380	546	1	+	181	20710.51	9.26	Alpha
**g80284.t1**	HlMADS47	001966F:73257:73870	501	1	-	166	18423.71	5.68	Alpha
**g76680.t1**	HlMADS48	001803F:4107:5760	1266	3	-	421	45520.49	9.34	Alpha
**g149874.t1**	HlMADS49	010116F:52407:53990	597	2	-	198	22248.4	8.78	Alpha
**g93699.t1**	HlMADS50	002616F:17167:18630	672	1	-	223	25007.1	5.87	Alpha
**g149872.t1**	HlMADS51	010116F:46656:47370	498	2	+	165	18540.17	5.11	Alpha
**g17586.t1**	HlMADS52	000193F:209997:216980	474	2	-	157	18031.71	6.52	Alpha
**g140155.t1**	HlMADS53	007017F:101746:102480	546	1	+	181	20852.1	6.32	Alpha
**g69465.t1**	HlMADS54	001521F:226037:229000	555	2	-	184	20509.14	5.82	Alpha
**g132514.t1**	HlMADS55	005766F:205237:206360	723	2	-	240	27195.68	8.72	Beta
**g123814.t1**	HlMADS56	004793F:82436:83690	987	2	+	328	36872.64	8.84	Beta
**g37290.t1**	HlMADS57	000578F:1005602:1008466	1256	2	+	395	44459.12	9.34	Beta
**g93696.t1**	HlMADS58	002616F:866:4050	708	3	+	235	26027.62	9.24	Alpha
**g20630.t1**	HlMADS59	000238F:870733:872867	759	2	+	252	28642.71	5.71	Alpha
**g71695.t1**	HlMADS60	001607F:395446:397250	591	2	+	196	22028.87	5.2	Alpha
**g34447.t1**	HlMADS61	000516F:40106:55690	1148	5	+	359	40443.46	8.84	Alpha
**g15081.t1**	HlMADS62	000156F:497105:498528	1100	3	-	343	39980.1	4.56	Beta
**g110780.t1**	HlMADS63	003683F:338687:340750	664	1	-	220	25301.54	4.76	Beta
**g32470.t1**	HlMADS64	000472F:1676:3320	1247	2	+	392	45421.25	4.55	Beta
**000453F.g47.t2**	HlMADS65	000453F:1097840:1106375	408	2	-	135	15911.6	9.69	SOC

Table S2. RNA-seq libraries used to hints AUGUSTUS

RNA-seq library	Tissues
**SRR10589377**	Leaf, bract and lupulin glands
**SRR10549511**	Leaf, bract and lupulin glands
**SRR10541757**	Leaf, bract and lupulin glands
**SRR10320795**	Non-floral Cascade RNA-seq (stem)
**SRR10320793**	Meristem-Cascade
**SRR10320791**	Leaf-Cascade
**SRR4242068**	Roots, sprouts, leaves, stems, flowers, cones
**ERR2040411**	Young leaves

Table S3. Gene Ontology terms of hop MADS-box genes

Genes	GO terms
**HlMADS02**	GO:0000977 GO:0000981 GO:0005634 GO:0008134 GO:0045944 GO:0046983
**HlMADS03**	GO:0000977 GO:0000981 GO:0005634 GO:0008134 GO:0045944 GO:0046983
**HlMADS04**	GO:0000977 GO:0003700 GO:0005634 GO:0045944 GO:0046983
**HlMADS05**	GO:0000977 GO:0003700 GO:0005634 GO:0045944 GO:0046983
**HlMADS06**	GO:0000977 GO:0000981 GO:0005634 GO:0008134 GO:0045944 GO:0046983
**HlMADS07**	GO:0000977 GO:0000981 GO:0005634 GO:0008134 GO:0045944 GO:0046983
**HlMADS08**	GO:0000977 GO:0000981 GO:0005634 GO:0008134 GO:0045944 GO:0046983
**HlMADS28**	GO:0000977 GO:0045944 GO:0046983
**HlMADS33**	GO:0000977 GO:0000981 GO:0005634 GO:0008134 GO:0010582 GO:0045944 GO:0046983
**HlMADS01**	GO:0000977 GO:0000981 GO:0005634 GO:0008134 GO:0010228 GO:0045944 GO:0046983 GO:0048364
**HlMADS31**	GO:0000977 GO:0000981 GO:0005634 GO:0008134 GO:0010228 GO:0045944 GO:0046983 GO:0048364
**HlMADS11**	GO:0000977 GO:0000981 GO:0005634 GO:0008134 GO:0010582 GO:0045944 GO:0046983
**HlMADS12**	GO:0000977 GO:0000981 GO:0005634 GO:0008134 GO:0010582 GO:0045944 GO:0046983
**HlMADS13**	GO:0000977 GO:0003700 GO:0005634 GO:0045944 GO:0046983
**HlMADS24**	GO:0000977 GO:0003700 GO:0005634 GO:0045944 GO:0046983
**HlMADS29**	GO:0000977 GO:0000981 GO:0005634 GO:0008134 GO:0016020 GO:0045944 GO:0046983 GO:0048481 GO:0090376
**HlMADS30**	GO:0000977 GO:0003700 GO:0005634 GO:0045944 GO:0046983 GO:0090376

**HlMADS32**	GO:0000977 GO:0000981 GO:0005634 GO:0008134 GO:0045944 GO:0046983 GO:0090376
**HlMADS19**	GO:0000977 GO:0003700 GO:0005634 GO:0045944 GO:0046983
**HlMADS20**	GO:0000977 GO:0003700 GO:0005634 GO:0045944 GO:0046983
**HlMADS09**	GO:0000977 GO:0003700 GO:0005634 GO:0045944 GO:0046983
**HlMADS10**	GO:0000977 GO:0003700 GO:0005634 GO:0045944 GO:0046983
**HlMADS18**	GO:0000977 GO:0003700 GO:0005634 GO:0045944 GO:0046983
**HlMADS15**	GO:0000977 GO:0003700 GO:0005634 GO:0008360 GO:0045595 GO:0045944 GO:0046983 GO:0048316 GO:0051302 GO:0080060 GO:0080155 GO:2000029
**HlMADS16**	GO:0000977 GO:0003700 GO:0005634 GO:0045944 GO:0046983
**HlMADS17**	GO:0000977 GO:0003700 GO:0005634 GO:0045944 GO:0046983
**HlMADS65**	GO:0000977 GO:0000981 GO:0005634 GO:0008134 GO:0010048 GO:0032501 GO:0045944 GO:0046983 GO:0048510
**HlMADS14**	GO:0003677 GO:0046983
**HlMADS22**	GO:0000981 GO:0000987 GO:0045944 GO:0046983
**HlMADS23**	GO:0000977 GO:0000981 GO:0005634 GO:0008134 GO:0045944 GO:0046983 GO:0048481
**HlMADS25**	GO:0000977 GO:0000981 GO:0005634 GO:0006357 GO:0008134 GO:0046983
**HlMADS34**	GO:0000977 GO:0000981 GO:0005634 GO:0008134 GO:0045944 GO:0046983
**HlMADS35**	GO:0000977 GO:0000981 GO:0005634 GO:0008134 GO:0045944 GO:0046983
**HlMADS36**	GO:0000977 GO:0045944 GO:0046983
**HlMADS37**	GO:0000977 GO:0045944 GO:0046983
**HlMADS38**	GO:0000977 GO:0000981 GO:0005634 GO:0008134 GO:0046983
**HlMADS39**	GO:0000977 GO:0000981 GO:0005634 GO:0008134 GO:0045944 GO:0046983
**HlMADS40**	GO:0000977 GO:0000981 GO:0005634 GO:0008134 GO:0045944 GO:0046983
**HlMADS41**	GO:0000977 GO:0000981 GO:0005634 GO:0008134 GO:0045944 GO:0046983
**HlMADS43**	GO:0000977 GO:0000981 GO:0005634 GO:0006357 GO:0008134 GO:0046983
**HlMADS44**	GO:0000977 GO:0000981 GO:0005634 GO:0008134 GO:0045944 GO:0046983
**HlMADS45**	GO:0000977 GO:0000981 GO:0005634 GO:0008134 GO:0045944 GO:0046983
**HlMADS46**	GO:0000977 GO:0000981 GO:0005634 GO:0008134 GO:0045944 GO:0046983
**HlMADS47**	GO:0000977 GO:0000981 GO:0005634 GO:0008134 GO:0045944 GO:0046983
**HlMADS48**	GO:0000977 GO:0000981 GO:0005634 GO:0006357 GO:0008134 GO:0046983
**HlMADS49**	GO:0000977 GO:0000981 GO:0005634 GO:0008134 GO:0045944 GO:0046983
**HlMADS50**	GO:0000976 GO:0046983
**HlMADS51**	GO:0000976 GO:0046983
**HlMADS52**	GO:0000976 GO:0046983
**HlMADS53**	GO:0000977 GO:0000981 GO:0005634 GO:0006357 GO:0008134 GO:0046983
**HlMADS54**	GO:0000977 GO:0000981 GO:0005634 GO:0006357 GO:0008134 GO:0046983
**HlMADS58**	GO:0000977 GO:0005634 GO:0045944 GO:0046983
**HlMADS59**	GO:0000976 GO:0046983
**HlMADS60**	GO:0000977 GO:0000981 GO:0005634 GO:0008134 GO:0046983
**HlMADS61**	GO:0000977 GO:0000981 GO:0005634 GO:0006357 GO:0008134 GO:0046983
**HlMADS26**	GO:0000977 GO:0000981 GO:0000987 GO:0005634 GO:0008134 GO:0045944 GO:0046983
**HlMADS21**	GO:0000981 GO:0000987 GO:0045944 GO:0046983
**HlMADS27**	GO:0003677 GO:0046983
**HlMADS42**	GO:0000977 GO:0000981 GO:0000987 GO:0005634 GO:0008134 GO:0008360 GO:0045595 GO:0045944 GO:0046983 GO:0048316 GO:0051302 GO:0080060 GO:0080155 GO:2000029
**HlMADS55**	GO:0003677 GO:0046983
**HlMADS56**	GO:0003677 GO:0046983
**HlMADS57**	GO:0003677 GO:0046983
**HlMADS62**	GO:0003677 GO:0046983
**HlMADS63**	GO:0003677 GO:0046983
**HlMADS64**	GO:0003677 GO:0046983

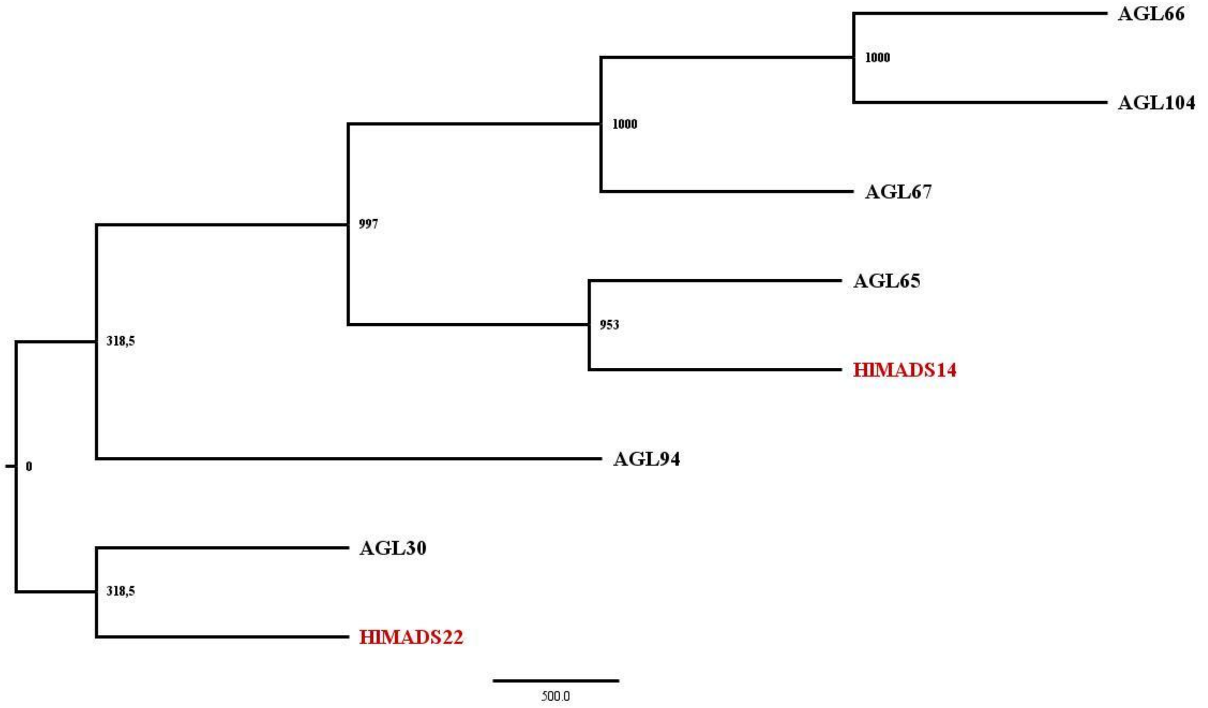

Figure S1. Phylogenetic tree of MIKC*-type MADS-box proteins of *H. lupulus* (2, red), Arabidopsis (6).

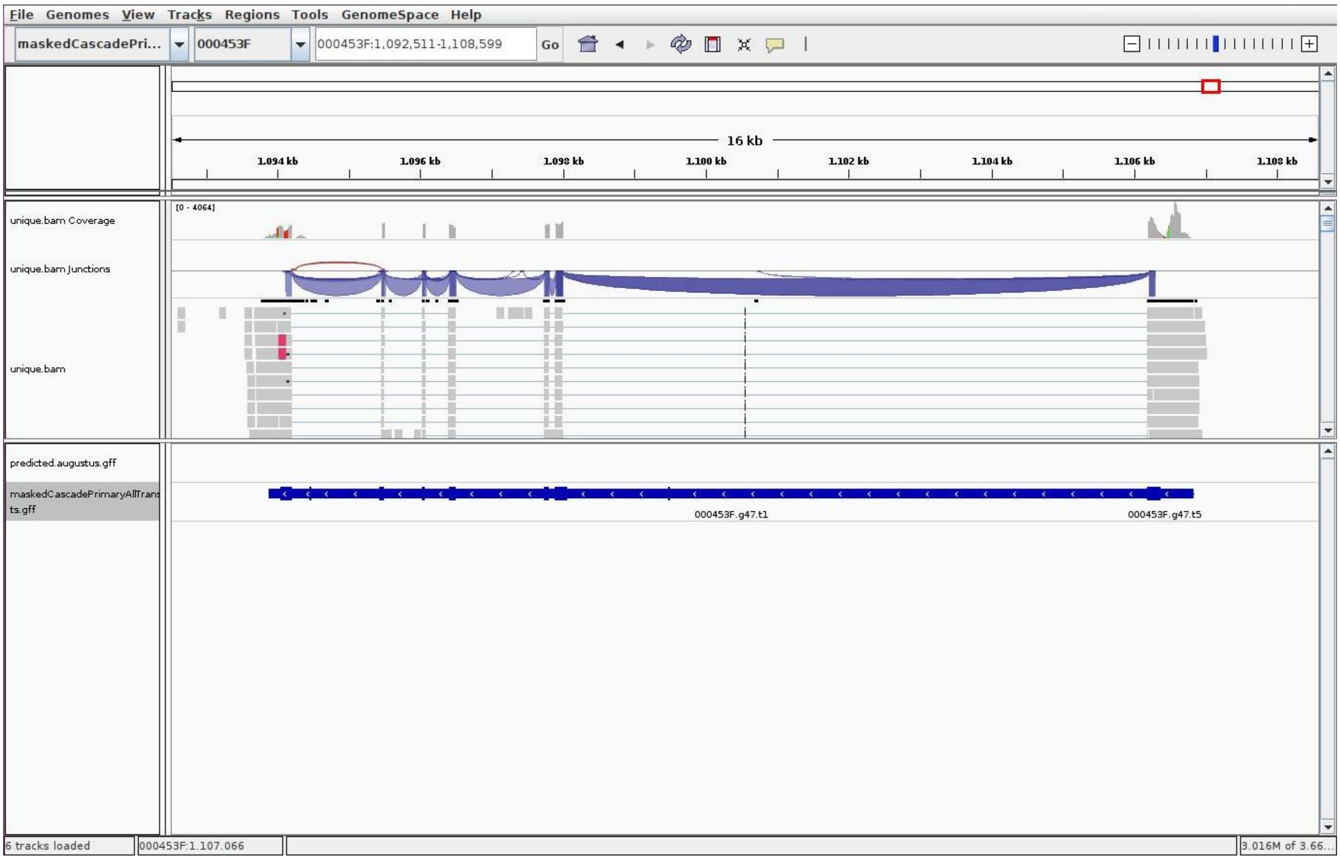

Figure S2. RNAseq reads on the gene *000453F.g47*.

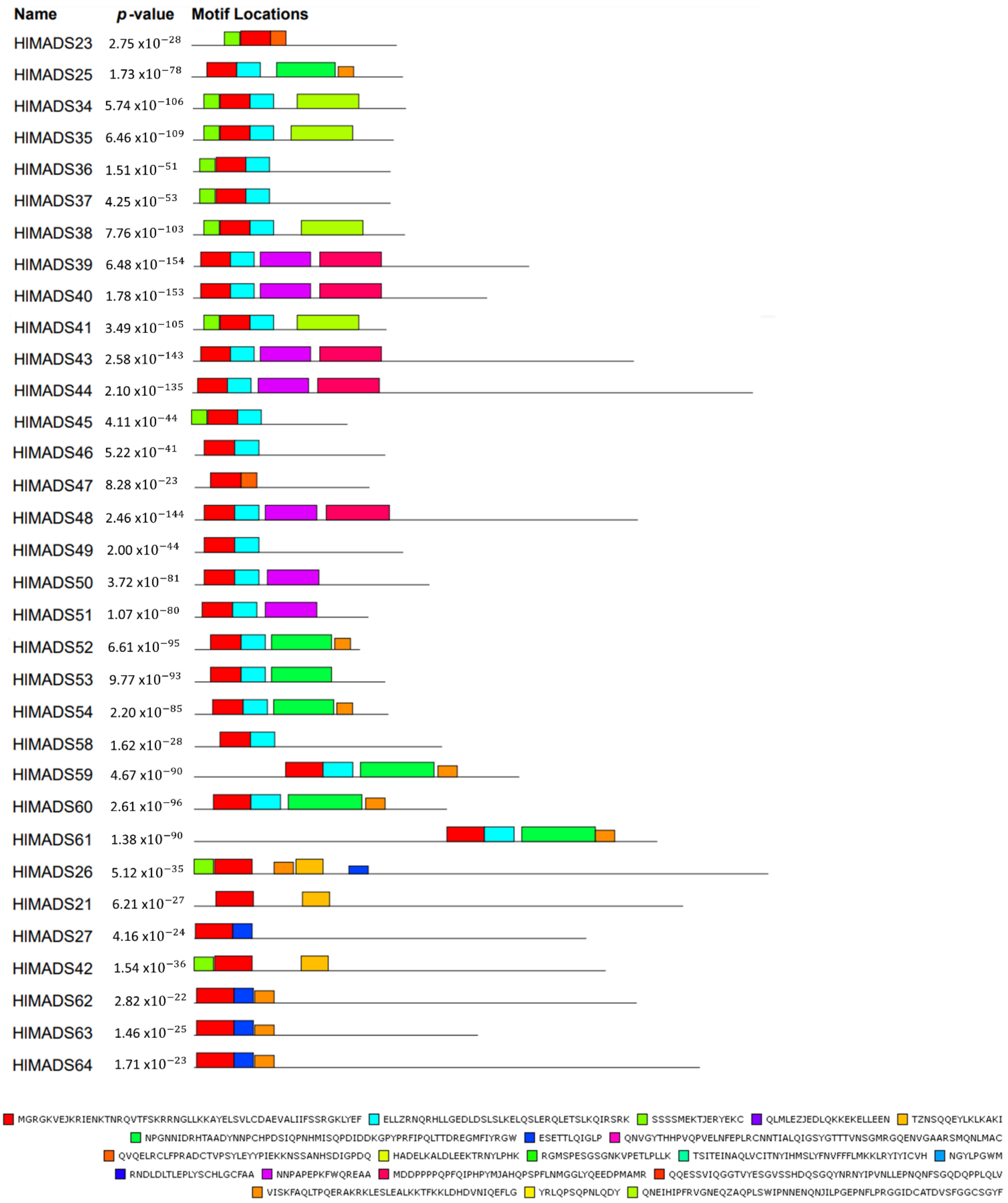

Figure S3. Motifs distribution of the hop MADS-box proteins. Protein sequences are represented by black lines, and the conserved motifs are represented by colored boxes. The motif consensus sequences are shown in the legend.

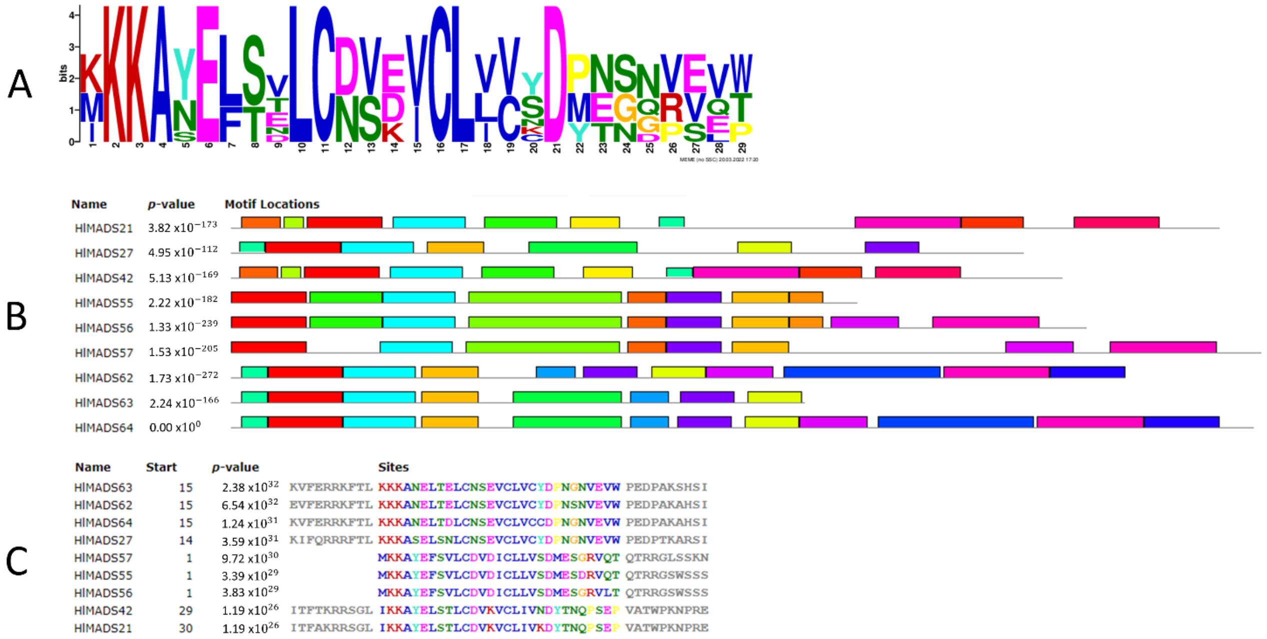

Figure S4. Motifs distribution of the hop MADS-box proteins of β-subfamily members. Protein sequences are represented by black lines, and the conserved motifs are represented by colored boxes. **A:** Logo of MADS-box domain. **B:** Motifs distribution of the hop MADS-box proteins of β-subfamily members. **C:** Amino acid residue sequences of selected MADS-box domains.

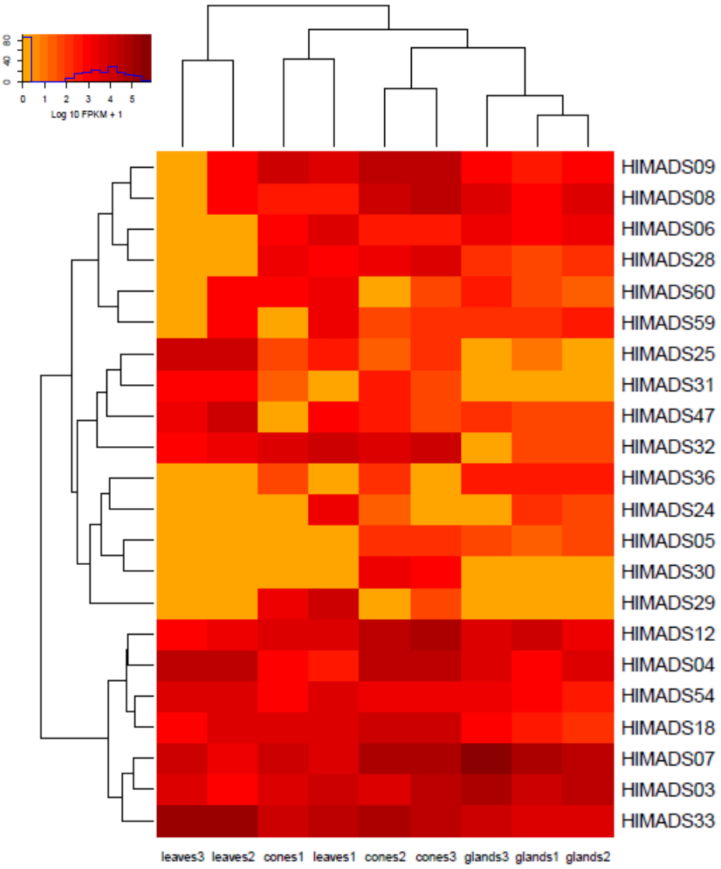

Figure S5. Expression profile of the hop MADS-box genes in three RNAseq libraries from cones, leaves, and glands.

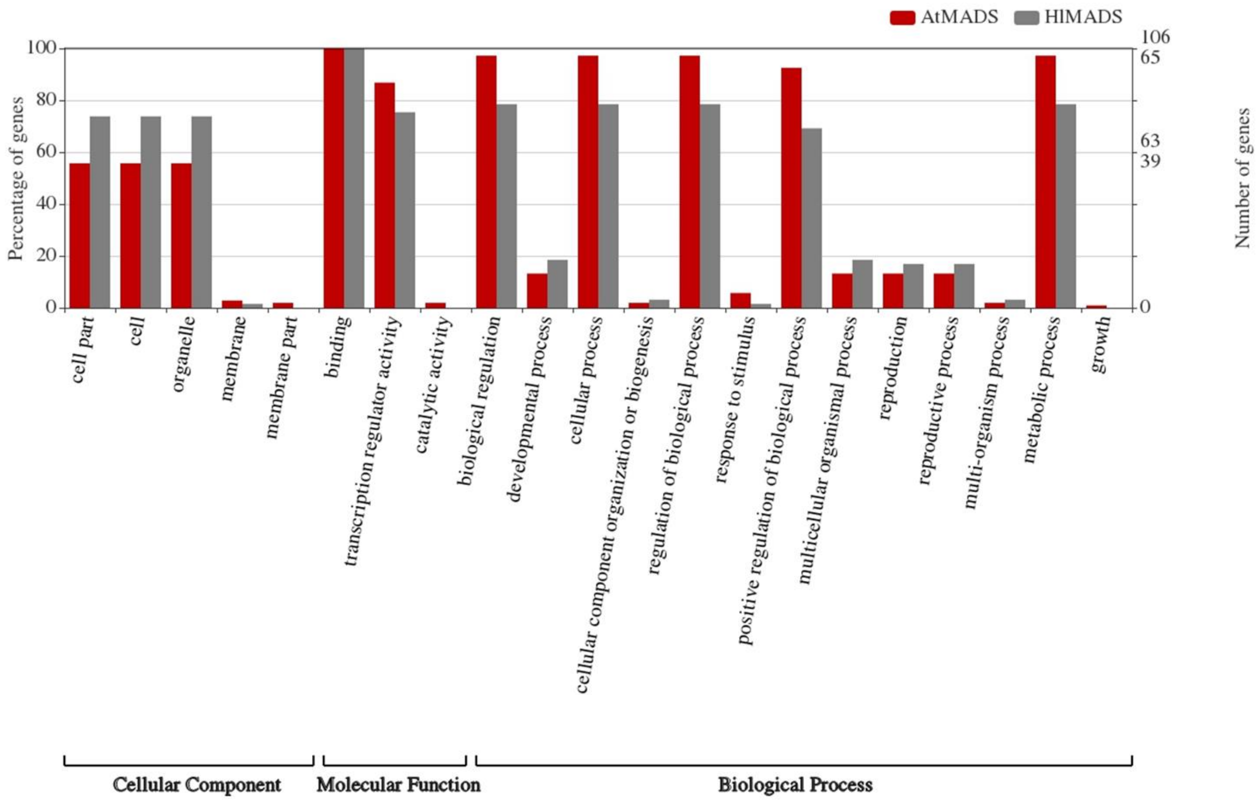

Figure S6. Gene Ontology classification of MADS-box genes in hop and *Arabidopsis*.

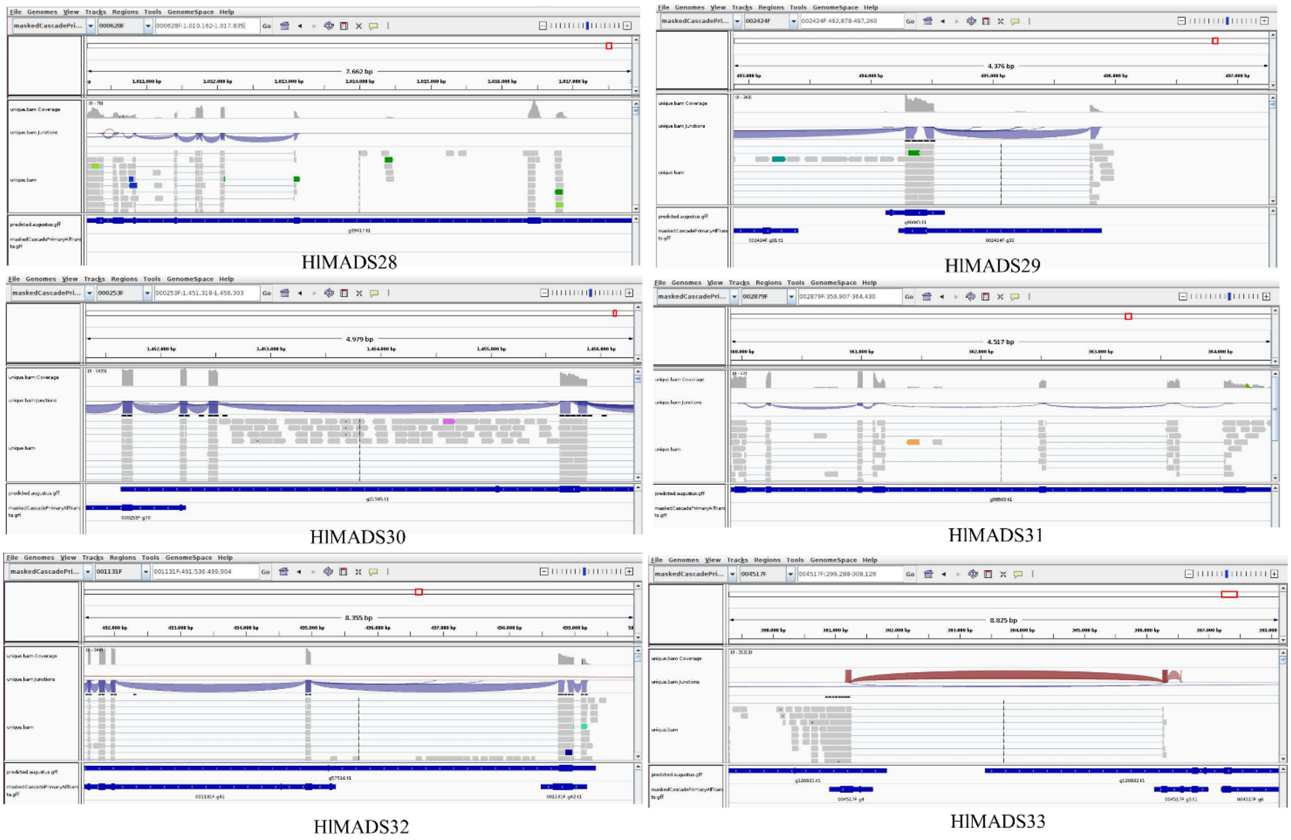

Figure S7. RNAseq reads that are uniquely mapped on 6 novel MADS-box genes (*HlMADS28-33*).
